# Overwintering of West Nile virus in a bird community with a communal crow roost

**DOI:** 10.1038/s41598-018-24133-4

**Published:** 2018-04-17

**Authors:** Diego Montecino-Latorre, Christopher M. Barker

**Affiliations:** 1 One Health Institute, School of Veterinary Medicine, University of California Davis, One Shields Ave., Davis, California 95616 USA; 2 Department of Pathology, Microbiology, and Immunology, School of Veterinary Medicine, University of California, Davis, CA 95616 USA

## Abstract

In temperate climates, transmission of West Nile virus (WNV) is detectable rarely during the coldest months (late fall through early spring), yet the virus has reappeared consistently during the next warm season. Several mechanisms may contribute to WNV persistence through winter, including bird-to-bird transmission among highly viremic species. Here we consider whether, under realistic scenarios supported by field and laboratory evidence, a winter bird community could sustain WNV through the winter in the absence of mosquitoes. With this purpose we constructed a deterministic model for a community of susceptible birds consisting of communally roosting crows, raptors and other birds. We simulated WNV introduction and subsequent transmission dynamics during the winter under realistic initial conditions and model parameterizations, including plausible contact rates for roosting crows. Model results were used to determine whether the bird community could yield realistic outbreaks that would result in WNV infectious individuals at the end of the winter, which would set up the potential for onward horizontal transmission into summer. Our findings strongly suggest that winter crow roosts could allow for WNV persistence through the winter, and our model results provide synthesis to explain inconclusive results from field studies on WNV overwintering in crow roosts.

## Introduction

West Nile virus (WNV; family *Flaviviridae*, genus *Flavivirus*) was introduced into New York in 1999^1^ and spread rapidly across the continent, reaching California in 2003^2^. The virus is maintained in transmission cycles between ornithophilic mosquitoes in the genus *Culex* and various passerine birds, with tangential transmission occurring when infectious mosquitoes bite humans^3–10^. The virus has caused over 40,000 human disease cases in the U.S., and more than 1,900 deaths^11^, and these numbers are underestimates^12^.

The intensity of WNV transmission is strongly seasonal due in part to influences of temperature. Warmer weather accelerates transmission by reducing the time for mosquito development and increasing rates of mosquito biting and viral replication^13–18^. Colder temperatures limit the reproduction of *Culex* mosquitoes and, along with shortening daylength, can induce some species to enter reproductive diapause^19–23^. As a consequence, in geographic locations with temperate climates such as California, mosquito-mediated arbovirus transmission declines to barely detectable levels during the winter season^13,24–26^.

Therefore, in bird communities exposed to extended cold seasons, it would be expected that WNV could fade out in the absence of other transmission mechanisms; however, the virus consistently reappears during the next warm season. WNV overwintering mechanisms supported by field or laboratory findings include vertical transmission to mosquitoes in winter rest, continued vector-bird transmission through the winter at low rates, direct transmission between avian hosts (predation scavenging and others pathways such as fecal-oral transmission) or recrudescence of viremia in chronically infected birds^19,20,26–44^. These mechanisms are not mutually exclusive and overwintering is almost certain to involve several of them. Of these possibilities, bird-to-bird transmission dynamics in winter avian communities remain poorly understood, and models are needed as a holistic framework to be reconciled with laboratory and field findings.

Within a winter bird community, American Crows (*Corvus brachyrhynchos*; hereafter “crows”) have the potential to play a significant role in WNV overwintering because this species is a highly competent host for WNV^39,40,45–52^. In winter, crows spend nights in communal roosts of thousands of birds flocking together^29,53,54^. Individuals living in large aggregations are likely to be in close proximity, therefore, crows are likely to have higher contact rates compared to other avian species^55,56^. Direct WNV transmission between crows has been demonstrated in the laboratory^39,40^, infected crows can shed the virus from the oral cavity^39^, and their feces can have high titers of WNV^50^. Field studies have reported that crows within a communal roost are frequently stained with feces of other crows and they exhibit preening behavior that could subject them to oral infection^29^, infected birds used the same roosts as healthy birds^29^ and WNV-positive dead crows have been recovered during cold periods when mosquitoes are not blood feeding^27,29,57^.

Consequently, in this study we used modeling to assess whether a realistic winter avian community consisting of crows, raptors and other birds can sustain WNV through the winter under plausible bird-to-bird transmission parameters in the absence of mosquitoes, while showing disease dynamics consistent with data from previous studies. We hypothesized that crow-to-crow transmission is the primary maintenance mechanism for WNV infection through the cold season, therefore, we initially identified the range of values for the crow-to-crow transmission parameter that yielded the largest fraction of realistic WNV outbreaks that resulted in viremic birds at the end of winter. We assessed the relevance of transmission among communally roosting crows and alternative WNV transmission pathways between birds (predation and scavenging), and finally, we evaluated the necessary conditions supporting realistic outbreaks and persistence of the virus through the winter across relevant parameter space. We discuss the plausibility of the selected crow-to-crow WNV transmission parameter values in nature, the coherence of model results with previous field studies, the relevance of other potential bird-to-bird transmission pathways for WNV overwintering, and the conditions supporting WNV overwintering in the bird community.

## Methods

### Study Population

Our study population consisted of the winter bird community of the University of California, Davis (UC Davis) (38.539975 N, 121.752187 W, Yolo County, California). The main campus has an area of ~7 km^2^ and it contains a well-documented crow roost with around 10,000 birds between November and March^29^. A previous study of this roost showed that crows are frequently stained with feces of other crows, crows exhibit preening behavior that could subject them to oral infection, WNV-infected and healthy crows are present in the roost, WNV-positive dead crows have been recovered during the cold season, and *Culex* mosquitoes are at very low abundance during this period^27,57^. The population of crows and other bird species are estimated yearly during a winter bird survey conducted during the third or fourth week of January by the UC Davis Museum of Wildlife and Fish Biology. The survey takes place in a single 24-hour period (midnight to midnight) in which over 40 walking observers systematically traverse all UC Davis campus. In this study, we used the mean counts from 2009–2014 annual censuses.

### Dynamical model

We constructed a deterministic, continuous-time model of WNV transmission within the study population. Bird species were classified into 3 types: (1) crows, which are competent hosts for WNV, have high contact rates, and scavenge on raptors and other birds; (2) raptors, which are competent hosts for WNV and prey on other birds, including crows; and (3) other birds, which could be targets of scavenging or predation by crows or raptors, respectively, but could not become infected. Crows and raptors were divided into 7 compartments: susceptible (S; birds that are not infected with WNV), exposed (E; birds that have been infected with WNV but are not yet infectious), acutely viremic (I_1_; birds that are viremic and infectious if contact occurs with competent S birds via predation, scavenging, or fecal shedding), fecal shedders (I_2_; birds that survive WNV viremia; however, they remain infectious to S birds through fecal-oral transmission as they continue shedding virus through feces), chronically infected (I_3_; birds that survived WNV viremia and they stop shedding WNV through feces, but maintain WNV in their organs and can be infectious if preyed upon), recovered (R; birds that clear WNV infection completely so that they are not infectious and remain permanently immune to new WNV infection) and dead (D; crows that died while in any other compartment and raptors that died while in the S, E, and R compartments). Dead raptors had an extra compartment: infectious dead (DI; raptors that die while in the I_1_, I_2_ and I_3_ compartments and are infectious if S crows scavenge them). The group of other birds were divided in 2 compartments: S and D, as we assumed that in absence of mosquitoes, they cannot get infected with WNV through another mechanism. Births were not modeled because the late fall and winter study period did not overlap the breeding season^58^.

The system of ordinary differential equations was as follows; for crows:

1 $$\frac{d{{\rm{S}}}_{{\rm{C}}}}{dt}=-\,{{\rm{\beta }}}_{{\rm{CC}}}{{\rm{S}}}_{{\rm{C}}}({{\rm{I}}}_{{\rm{C}}1}+{{\rm{I}}}_{{\rm{C}}2})-{{\rm{\alpha }}}_{{\rm{CB}}}{{\rm{n}}}_{{\rm{C}}}{{\rm{p}}}_{{\rm{CB}}}\frac{{{\rm{S}}}_{{\rm{C}}}}{{{\rm{S}}}_{{\rm{C}}}+{{\rm{E}}}_{{\rm{C}}}+{{\rm{I}}}_{{\rm{C}}2}+{{\rm{I}}}_{{\rm{C}}3}+{{\rm{R}}}_{{\rm{C}}}}{{\rm{DI}}}_{{\rm{R}}}-{{\rm{\mu }}}_{{\rm{C}}}{{\rm{S}}}_{{\rm{C}}}$$

2 $$\frac{d{{\rm{E}}}_{{\rm{C}}}}{dt}={{\rm{\beta }}}_{{\rm{CC}}}{{\rm{S}}}_{{\rm{C}}}({{\rm{I}}}_{{\rm{C}}1}+{{\rm{I}}}_{{\rm{C}}2})+{{\rm{\alpha }}}_{{\rm{CB}}}{{\rm{n}}}_{{\rm{C}}}{{\rm{p}}}_{{\rm{CB}}}\frac{{{\rm{S}}}_{{\rm{C}}}}{{{\rm{S}}}_{{\rm{C}}}+{{\rm{E}}}_{{\rm{C}}}+{{\rm{I}}}_{{\rm{C}}2}+{{\rm{I}}}_{{\rm{C}}3}+{{\rm{R}}}_{{\rm{C}}}}{{\rm{DI}}}_{{\rm{R}}}-{\epsilon }_{{\rm{C}}}{{\rm{E}}}_{{\rm{C}}}-{{\rm{\mu }}}_{{\rm{C}}}{{\rm{E}}}_{{\rm{C}}}$$

3 $$\frac{d{{\rm{I}}}_{{\rm{C}}1}}{dt}={\epsilon }_{{\rm{C}}}{{\rm{E}}}_{{\rm{C}}}-{{\rm{\gamma }}}_{{\rm{C}}1}{{\rm{I}}}_{{\rm{C}}1}-{{\rm{\mu }}}_{{\rm{C}}}{{\rm{I}}}_{{\rm{C}}1}$$

4 $$\frac{d{{\rm{I}}}_{{\rm{C}}2}}{dt}=(1-{{\rm{\rho }}}_{{\rm{C}}}){{\rm{\gamma }}}_{{\rm{C}}1}{{\rm{I}}}_{{\rm{C}}1}-{{\rm{\gamma }}}_{{\rm{C}}2}{{\rm{I}}}_{{\rm{C}}2}-{{\rm{\mu }}}_{{\rm{C}}}{{\rm{I}}}_{{\rm{C}}2}$$

5 $$\frac{d{{\rm{I}}}_{{\rm{C}}3}}{dt}={{\rm{\lambda }}}_{{\rm{C}}}{{\rm{\gamma }}}_{{\rm{C}}2}{{\rm{I}}}_{{\rm{C}}2}-{{\rm{\gamma }}}_{{\rm{C}}3}{{\rm{I}}}_{{\rm{C}}3}-{{\rm{\mu }}}_{{\rm{C}}}{{\rm{I}}}_{{\rm{C}}3}$$

6 $$\frac{d{{\rm{R}}}_{{\rm{C}}}}{dt}=(1-{{\rm{\lambda }}}_{{\rm{C}}}){{\rm{\gamma }}}_{{\rm{C}}2}{{\rm{I}}}_{{\rm{C}}2}+{{\rm{\gamma }}}_{{\rm{C}}3}{{\rm{I}}}_{{\rm{C}}3}-{{\rm{\mu }}}_{{\rm{C}}}{{\rm{R}}}_{{\rm{C}}}$$

7 $$\frac{d{{\rm{D}}}_{{\rm{C}}}}{dt}=({{\rm{\mu }}}_{{\rm{C}}}-{{\rm{\alpha }}}_{{\rm{RC}}})({{\rm{S}}}_{{\rm{C}}}+{{\rm{E}}}_{{\rm{C}}}+{{\rm{I}}}_{{\rm{C}}1}+{{\rm{I}}}_{{\rm{C}}2}+{{\rm{I}}}_{{\rm{C}}3}+{{\rm{R}}}_{{\rm{C}}})+{{\rm{\rho }}}_{{\rm{C}}}{{\rm{\gamma }}}_{{\rm{C}}1}{{\rm{I}}}_{{\rm{C}}1}-{{\rm{\tau }}D}_{{\rm{C}}}$$

for raptors:

8 $$\frac{d{{\rm{S}}}_{{\rm{R}}}}{dt}=-\,{{\rm{\alpha }}}_{{\rm{RC}}}{{\rm{p}}}_{{\rm{RC}}}({{\rm{I}}}_{{\rm{C}}1}+{{\rm{I}}}_{{\rm{C}}2}+{I}_{{\rm{C}}3})\frac{{{\rm{S}}}_{{\rm{R}}}}{{{\rm{S}}}_{{\rm{R}}}+{{\rm{E}}}_{{\rm{R}}}+{{\rm{I}}}_{{\rm{R}}1}+{{\rm{I}}}_{{\rm{R}}2}+{{\rm{I}}}_{{\rm{R}}3}+{{\rm{R}}}_{{\rm{R}}}}-{{\rm{\mu }}}_{{\rm{R}}}{{\rm{S}}}_{{\rm{R}}}$$

9 $$\frac{d{{\rm{E}}}_{{\rm{R}}}}{dt}={{\rm{\alpha }}}_{{\rm{RC}}}{{\rm{p}}}_{{\rm{RC}}}({{\rm{I}}}_{{\rm{C}}1}+{{\rm{I}}}_{{\rm{C}}2}+{I}_{{\rm{C}}3})\frac{{{\rm{S}}}_{{\rm{R}}}}{{{\rm{S}}}_{{\rm{R}}}+{{\rm{E}}}_{{\rm{R}}}+{{\rm{I}}}_{{\rm{R}}1}+{I}_{{\rm{R}}2}+{{\rm{I}}}_{{\rm{R}}3}+{{\rm{R}}}_{{\rm{R}}}}-{\epsilon }_{{\rm{R}}}{{\rm{E}}}_{{\rm{R}}}-{{\rm{\mu }}}_{{\rm{R}}}{{\rm{E}}}_{{\rm{R}}}$$

10 $$\frac{d{{\rm{I}}}_{{\rm{R}}1}}{dt}={\epsilon }_{{\rm{R}}}{{\rm{E}}}_{{\rm{R}}}-{{\rm{\gamma }}}_{{\rm{R}}1}{{\rm{I}}}_{{\rm{R}}1}-{{\rm{\mu }}}_{{\rm{R}}}{{\rm{I}}}_{{\rm{R}}1}$$

11 $$\frac{d{{\rm{I}}}_{{\rm{R}}2}}{dt}=(1-{{\rm{\rho }}}_{{\rm{R}}}){{\rm{\gamma }}}_{{\rm{R}}1}{{\rm{I}}}_{{\rm{R}}1}-{{\rm{\gamma }}}_{{\rm{R}}2}{{\rm{I}}}_{{\rm{R}}2}-{{\rm{\mu }}}_{{\rm{R}}}{{\rm{I}}}_{{\rm{R}}2}$$

12 $$\frac{d{{\rm{I}}}_{{\rm{R}}3}}{dt}={{\rm{\lambda }}}_{{\rm{R}}}{{\rm{\gamma }}}_{{\rm{R}}2}{{\rm{I}}}_{{\rm{R}}2}-{{\rm{\gamma }}}_{{\rm{R}}3}{{\rm{I}}}_{{\rm{R}}3}-{{\rm{\mu }}}_{{\rm{R}}}{{\rm{I}}}_{{\rm{R}}2}$$

13 $$\frac{d{{\rm{R}}}_{{\rm{R}}}}{dt}=(1-{{\rm{\lambda }}}_{{\rm{R}}}){{\rm{\gamma }}}_{{\rm{R}}2}{{\rm{I}}}_{{\rm{R}}2}+{{\rm{\gamma }}}_{{\rm{R}}3}{{\rm{I}}}_{{\rm{R}}3}-{{\rm{\mu }}}_{{\rm{R}}}{{\rm{R}}}_{{\rm{R}}}$$

14 $$\frac{d{{\rm{D}}}_{{\rm{R}}}}{dt}={{\rm{\mu }}}_{{\rm{R}}}({{\rm{S}}}_{{\rm{R}}}+{{\rm{E}}}_{{\rm{R}}}+{{\rm{R}}}_{{\rm{R}}})-{{\rm{\tau }}}_{{\rm{R}}}{{\rm{D}}}_{{\rm{R}}}-{{\rm{\alpha }}}_{{\rm{CB}}}{{\rm{n}}}_{{\rm{C}}}({{\rm{S}}}_{{\rm{C}}}+{{\rm{E}}}_{{\rm{C}}}+{{\rm{I}}}_{{\rm{C}}2}+{{\rm{I}}}_{{\rm{C}}3}+{{\rm{R}}}_{{\rm{C}}}){{\rm{D}}}_{{\rm{R}}}$$

15 $$\frac{d{{\rm{DI}}}_{{\rm{R}}}}{dt}={{\rm{\rho }}}_{{\rm{R}}}{{\rm{\gamma }}}_{{\rm{R}}1}{{\rm{I}}}_{{\rm{R}}1}+{{\rm{\mu }}}_{{\rm{R}}}({{\rm{I}}}_{{\rm{R}}1}+{{\rm{I}}}_{{\rm{R}}2}+{{\rm{I}}}_{{\rm{R}}3})-{{\rm{\tau }}}_{{\rm{R}}}{{\rm{DI}}}_{{\rm{R}}}-{{\rm{\alpha }}}_{{\rm{CB}}}{{\rm{n}}}_{{\rm{C}}}({{\rm{S}}}_{{\rm{C}}}+{{\rm{E}}}_{{\rm{C}}}+{{\rm{I}}}_{{\rm{C}}2}+{{\rm{I}}}_{{\rm{C}}3}+{{\rm{R}}}_{{\rm{C}}}){{\rm{DI}}}_{{\rm{R}}}$$

and for other birds:

16 $$\frac{d{{\rm{S}}}_{{\rm{O}}}}{dt}=-\,{{\rm{\mu }}}_{{\rm{O}}}{{\rm{S}}}_{{\rm{O}}}$$

17 $$\frac{d{{\rm{D}}}_{{\rm{O}}}}{dt}=({{\rm{\mu }}}_{O}-{{\rm{\alpha }}}_{{\rm{RO}}}){{\rm{S}}}_{{\rm{O}}}-{{\rm{\tau }}}_{{\rm{O}}}{{\rm{D}}}_{{\rm{O}}}-{{\rm{\alpha }}}_{{\rm{CB}}}{{\rm{n}}}_{{\rm{C}}}({{\rm{S}}}_{{\rm{C}}}+{{\rm{E}}}_{{\rm{C}}}+{{\rm{I}}}_{{\rm{C}}2}+{{\rm{I}}}_{{\rm{C}}3}+{{\rm{R}}}_{{\rm{C}}}){{\rm{D}}}_{{\rm{O}}}$$

**Figure 1:**
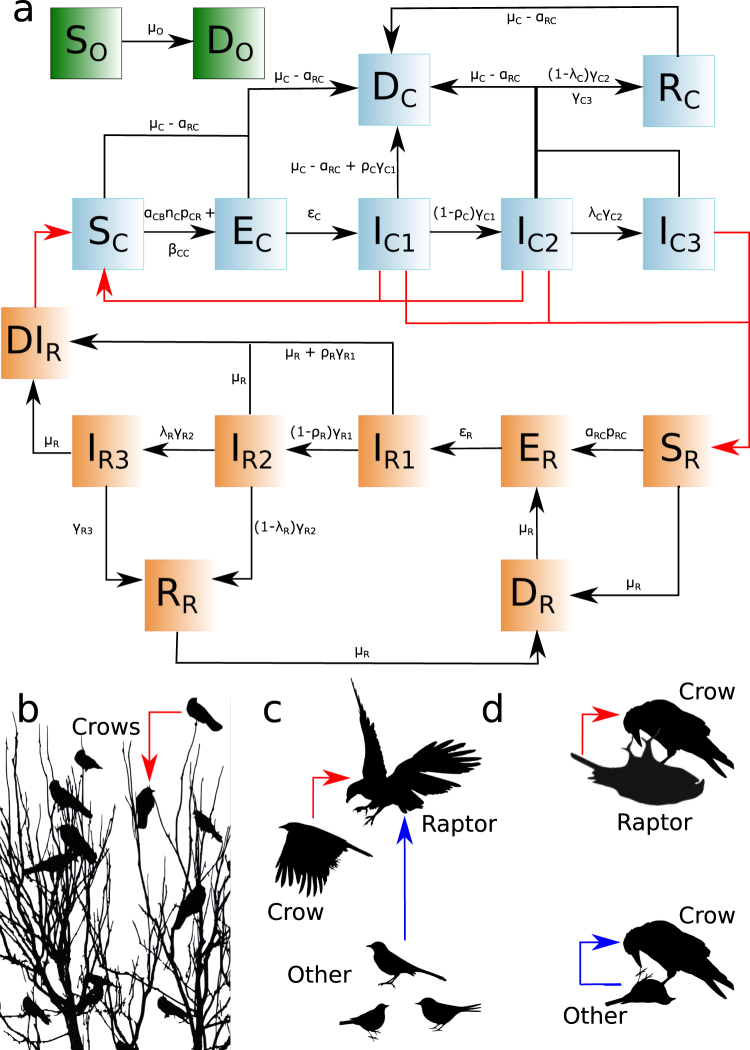
Model summary. (**a**) Bird compartments and parameters determining compartment transitions. The green, light blue and orange boxes correspond to O, C and R compartments, respectively. Black arrows show movement of C, R and O among compartments, while red arrows depict routes of WNV transmission. (**b**) Interactions among crows in the roost. (**c**) Predation of raptors on crows and other birds. (**d**) Scavenging of crows on carcasses of raptors and other birds. In (**b**), (**c**) and (**d**) the red arrows shows interactions that may involve WNV transmission. Blue arrow shows interactions not involving WNV transmission. Credits: Crows roosting in 1b: Diego Montecino-Latorre; crow flying in 1c: Emilian Robert Vicol and Bob Comix (http://www.supercoloring.com/silhouettes/crows; published under a CCBY SA license); raptor flying in 1c: (https://www.vecteezy.com/vector-art/94660-free-eagle-silhouette-vector); other bird in bottom right of 1c: Russell Murphy (http://animalsclipart.com/small-bird-silhouette); other bird below the blue arrow in 1c: Matthew Townsend and Bob Comix (http://www.supercoloring.com/silhouettes/mockingbird; published under a CCBY SA license); other bird in the bottom left of 1c and dead other bird in 1d: Wanda Butler (http://animalsclipart.com/bird-silhouette); crow scavenging in 1d: https://openclipart.org/detail/259888/raven-silhouette-2); and dead raptor in 1d: Natalia Duque.

The subscripts C, R and O for the compartments refers to crows, raptors and other birds, respectively. A full summary of model parameters is shown in Table 1, while bird states, parameters, and interactions are summarized in Fig. 1.

**Table 1:** Parameters used in the model with the values used and their definition.

Symbol	Parameter Definition	Value	Reference
β_CC_	Daily per capita WNV crow-to-crow transmission rate	Initially 2.14*10^–9^–2	^75,76^
E_C_	The initial number of WNV-infected crows at the start of winter	1–50	*
ɑ_CB_	Daily per capita rate of crows scavenging upon bird carrion	0.001–0.01	*
ɑ_RC_	Daily per capita rate of raptors feeding upon crows	μ_C_* pred_RC_	*
ɑ_RO_	Daily per capita rate of raptors feeding upon other birds	μ_O_* pred_RO_	*
pred_RC_	Proportion of crow daily mortality rate due raptor predation	0.01–0.2	*
pred_RO_	Proportion of other birds daily mortality rate due raptor predation	0.05–0.2	^58,94–99^
n_C_	Number of crows scavenging from a single bird carcass	1–20	*
p_CB_	Probability that S_C_ scavenging DI_R_ gets infected with WNV	0.7–0.9	^39^
p_RC_	Probability that S_R_ feeding upon I_C1_, I_C2_ or I_C3_ gets infected with WNV	0.15–0.5	^39,59^
ε_C_	Daily per capita rate E_C_ become acutely viremic: I_C1_	0.333–1	^39,46,50,51,63^
ε_R_	Daily per capita rate E_R_ become acutely viremic: I_R1_	0.333–1	^39,59^
γ_C1_	Daily per capita rate I_C1_ clear WNV viremia	0.2–0.333	^39,50^
γ_C2_	Daily per capita rate I_C2_ clear WNV fecal shedding after the viremia	0.111–0.167	^50^
γ_C3_	Daily per capita rate I_C3_ clear WNV chronic infection	0.011–0.018	^41,42,44^
γ_R1_	Daily per capita rate I_R1_ clear WNV viremia	0.2–0.333	^39,59^
γ_R2_	Daily per capita rate I_R2_ clear WNV fecal shedding	0.2–0.333	^39,59^
γ_R3_	Daily per capita rate I_R3_ clear WNV chronic infection	0.011–0.018	^41,42,44^
ρ_C_	Probability that I_C1_ die due WNV at the end of the acute viremic period	0.9–1	^39,40,46,47,49,50^
ρ_R_	Probability that I_R1_ die due WNV at the end of the acute viremic period	0.01–0.05	^39,59^
λ_C_	Probability that I_C2_ will remain infected in their organs	0.15–0.35	^42^
λ_R_	Probability that I_R2_ will remain infected in their organs	0.2–0.7	^39,42,59^
μ_C_	Daily per capita mortality rate for crows	0.0003–0.0005	^100^
μ_R_	Daily per capita mortality rate for raptors	0.0005–0.0009	^101–104^
μ_O_	Daily per capita mortality rate for other birds	0.0009–0.0027	^105–135^
γ_C_	Daily per capita rate of D_C_ elimination from the system through decomposition	0.2–0.333	^66,67^
τ_R_	Daily per capita rate of D_R_ and DI_R_ decomposition	0.2–0.333	^66,67^
τ_O_	Daily per capita rate of D_O_ decomposition	0.2–0.333	^66,67^

Parameter values for raptors and other birds are the weighted estimate from values reported in previous studies. Weight was given according to the population raptor and other birds species with reported values represented.

Susceptible crows (S_C_) became E_C_ after an infectious contact with I_C1_ and I_C2_^39,40^ at daily per capita transmission rate β_CC_, and/or after scavenging upon DI_R_ at ɑ_CB_*n_C_*p_CB_ daily rate^39,40,59–61^, where ɑ_CB_ is the daily per capita rate of crows scavenging upon bird carrion, n_C_ is the number of crows that scavenge upon a single raptor or other bird carcass, and *p*_CB_ is the WNV transmission probability from WNV-infected bird carrion to scavenging S_C_. We parameterized predator-prey infectious disease transmission as recommended in past research^62^. The rate at which crows scavenged upon DI_R_ was proportional to their availability with respect to other carcasses (D_R_ and D_O_). We assumed that crows did not feed upon dead conspecifics because this behavior has not been observed in the study population. The E_C_ became I_C1_ at rate ε_C_^39,46,50,51,63^, and died due to WNV with a probability ρ_C_ at the end of the acutely viremic period. Most I_C1_ died following WNV viremia^39,40,45–52^; however, surviving individuals cleared the viremia at rate γ_C1_ and moved to I_C2_. The I_C2_ remained infectious as they continued shedding WNV through feces^39,50^ and left this compartment at rate γ_C2_ moving to one of 2 compartments: I_C3_ or R_C_ with probabilities λ_C_ and 1-λ_C_, respectively^41,42,44^, where λ_C_ is the probability of becoming chronically infected. The I_C3_ individuals retained WNV in their organs^39,51,63^, and could transmit the disease if fed upon by susceptible raptors (S_R_, see below), while R_C_ cleared WNV infection and retained immunity for life^42,64,65^. Finally, I_C3_ cleared the chronic infection at rate γ_C3_ and moved to R_C_^41,42,44^. All crows moved to D_C_ according to the proportion of the expected mortality rate of crows, μ_C_, not explained by raptor predation: μ_C_- α_RC_, where α_RC_ is the predation rate of raptors upon crows and equals μ_C_* pred_RC_. Here, pred_RC_ is the proportion of μ_C_ explained by raptor predation over crows. D_C_ decomposed and were removed from the system at rate γ_C_^66,67^.

The S_R_ moved to E_R_ when preying upon the I_C1_, I_C2_ and I_C3_ fractions of all live crows, N_C_^39,59^. This predation happened at α_RC_*p_RC_ rate, where *p*_RC_ is the WNV transmission probability from infected crows to raptors. We parameterized predator-prey infectious disease transmission as previously mentioned^62^. We assumed all raptors could prey on other birds because WNV-infected raptors continue to feed^59^. Individuals in the E_R_ compartment became acutely viremic, I_R1_, at rate ε_R_^39,59^. The WNV-induced mortality at the end of the viremia for the raptor species present in our study area, ρ_R_, is lower than ρ_C_^39,59,60,68^. Similar to crows, raptors surviving I_R1_ move to I_R2_, at rate γ_R1_^39,59^, while I_R2_ recovered from shedding at rate γ_R2_^39,59^ and moved into one of the two compartments, I_R3_ or R_R_, with probabilities λ_R_ and 1-λ_R_, respectively^41,42,44,59^, where λ_R_ is the probability that raptors become chronically infected. Raptors in I_R3_ cleared the infection and moved into the R_R_ compartment at rate γ_R3_^41,42,44^. All raptors died at rate μ_R_, and those that died in non-infectious compartments S_R_, E_R_ and R_R_ moved to D_R_. Raptors that died in the I_R1_, I_R2_ and I_R3_ compartments became infectious after death (DI_R_) to scavenging S_C_. We assumed DI_R_ remained infectious until decomposed or consumed^39,69^. Finally, D_R_ and DI_R_ rot and were removed from the system at rate τ_R_^66,67^, and also by scavenging crows at rate ɑ_CB_*n_C_. Individuals in the I_C1_ compartment did not scavenge due behavioral changes as result of WNV illness^39^.

Other birds remained uninfected (S_O_) and died at rate μ_O_. Dead other birds that remained in the system, D_O_, arose at rate μ_O_ - ɑ_RO_ and included those that died from causes other than raptor predation. Here, α_RO_ is the predation rate of raptors upon other birds and equals μ_O_* pred_RO_, where pred_RO_ is the proportion of μ_O_ explained by raptor predation over other birds. D_O_ decomposed and were removed from the system at rate τ_O_^66,67^, and also by crows scavenging upon them at rate ɑ_CB_*n_C_.

Crow-to-crow transmission in the roost was modeled as density-dependent, implying that the transmission rate among crows increased linearly with the number of crows per unit area^70^. We also assumed that crow-to-crow transmission occurred exclusively when roosting, that the area used by the UC Davis bird community and the crow roost remained constant over the time period simulated^1114^, that the bird community is closed during winter after the initial introduction of WNV, and that the course of the disease in birds infected through bird-to-bird transmission follows that of mosquito-bird transmission^39^.

### Simulations

We simulated the introduction of E_C_ into a completely susceptible study population on November 1 (time 0), which represents the most permissive scenario for WNV transmission at that time of year. After introduction, we ran the model for 151 days ending March 31, which was considered late enough for mosquito-bird transmission cycles to take over as the primary mechanism of viral amplification into spring and summer and because crows stop roosting communally by this time^29^.

In order to account for uncertainty about model parameters, we constructed 300 parameter sets by Latin Hypercube Sampling (LHS) from the ranges defined in Table 1 (except β_CC_, see next paragraph) using the ‘lhs’ package^71^ in R^72^, with κ_*i*_~Unif(κ_*imin*_, κ_*imax*_) where **κ** is the vector of 26 parameters in the model. We used 300 parameter sets for our deterministic simulations following recommendations for LHS of 10 samples per parameter assessed^73^.

### Finding the crow-to-crow daily WNV transmission rate (β_CC_) range causing the largest proportion of realistic WNV outbreaks

We used iterative sampling of the parameter space to determine the range for β_CC_ that would result in a high probability of WNV persistence through the winter whilst remaining realistic. Specifically, we searched the β_CC_ range for values that maximized the number of 300 simulations causing: a) at least 15 infected birds at the end of the winter, which we assumed to be a number large enough to avoid stochastic fadeout of WNV during onward bird-mosquito amplification into the warmer season, b) at least 67% of the original crow population living at the end of the winter, because it is not expected to lose more than one third of these birds after WNV initial introduction in a completely susceptible crow population^74^, c) less than 95% of the original crow population as this is the median crow population at the end of the winter when no WNV is introduced in our model, and d) less than 200 dead birds on any particular day of the study period because larger die-offs at the spatial scale of this study have not been observed and would have been unlikely to occur unnoticed. Therefore, we focused on the following three outcomes of interest (hereafter ‘OoI’): the number of crows at the end of winter, the number of infectious birds at the end of the winter, and the maximum number of dead birds at any given day during the simulation period.

We defined an initial β_CC_ range of [2.14*10^−9^–2] infectious contacts per crow per day. The lower bound for this range was chosen because it matches the avian influenza transmission parameter estimated for waterfowl^75^, which we expected to be lower than the contact rate for gregarious crows. The upper bound of 2 was chosen because it is closer to the fecal-oral transmission rate for *Campylobacter jejuni* in chicken flocks (2.4, bacterial but fecal-oral transmission) previously estimated^76^. We regarded β_CC_ >2 as unlikely to occur in free-ranging populations of roosting crows.

The initial β_CC_ range was partitioned in ranges defined by minβ_CC_, maxβ_CC_ and its quartiles _*q*1_β_CC_ = [minβ_CC_,…,Q_1_β_CC_], _*q*2_β_CC_ = (Q_1_β_CC_, Q_2_β_CC_], _*q*3_β_CC_ = (Q_2_β_CC_, Q_3_β_CC_] and _*q*4_β_CC_ = (Q_3_β_CC_, maxβ_CC_]. With each range we generated by LHS the 300 sets of unique parameter values. We ran the model with each parameter set, recording the proportion of simulations that were within our bounds for realism for the three OoI, which corresponded to ‘realistic simulations.’ We selected the _*qj*_β_CC_ that yielded the greatest proportion of realistic simulations, and then divided this selected _*qj*_β_CC_ again as previously explained. We continued this selection process, subdividing selected ranges and running sets of 300 simulations at each step until we found the values that caused the largest proportion of realistic simulations in a range.

### Relevance of transmission among communally roosting crows and alternative WNV transmission pathways between birds such as predation and scavenging

Once the β_CC_ range was found, we conducted a global sensitivity analysis as recommended^77^ with the purpose to find those significant parameters for which the OoI were sensitive. We used the results from 300 LHS draws to estimate the partial rank correlation coefficient (PRCC) of each parameter in the model with respect to each OoI. We tested the null hypothesis that there was no correlation between each parameter and the OoI. Monotonicity of the relationship between κ_*i*_ and the number of crows, the number of infectious birds at the end of the winter, and the maximum number of dead birds at any given day was assessed graphically. In consequence, a separate set of parameters with significant PRCCs for at least one OoI was identified (denoted as **ϴ**; therefore, **ϴ** U **ϴ**’ = **κ**, where **κ** is the complete set of 26 parameters in the model).

### Conditions supporting realistic outbreaks and infectious birds at the end of the winter across the parameter space

We explored the OoI across the space of selected β_CC_ range and the parameters in **ϴ**, by partitioning their ranges as explained previously. Using LHS we obtained 100 unique values for β_CC_ as well as for each parameter in **ϴ** from the corresponding _*qj*_β_CC_ and _*qj*_**ϴ**_*k*_ ranges, while the remaining significant parameters in **ϴ**, and the non-significant parameters in **ϴ**’ were assigned the mean value of their original range (Table 1). We ran the model with each set of parameters and calculated the proportion of simulations that fulfilled our thresholds for realism. From these results we evaluated the conditions across the parameter space supporting WNV overwintering in the avian community in the absence of vectors.

### Data availability

Data generated and analyzed during the current study are available in the figshare repository^78^.

## Results

### Study Population

The bird community in this study initially consisted of 9,952 crows, 112 raptors, and 12,409 other birds.

### Finding the crow-to-crow daily WNV transmission rate (β_CC_) range causing the largest proportion of realistic WNV outbreaks

The broadest *a priori* ranges of parameters resulted in no realistic outbreaks. For these simulations, a median of 13 (range: 0–54) infectious birds and 439 (range: 0–876) total living crows remained at the end of the winter, while the median peak daily number of dead birds was 2,921 (range: 1,970–3,985). These simulations resulted in very rapid spread of WNV through the crow population, causing extremely high mortality and exhausting most susceptibles before the study period was over. However, after 20 cycles of parameter selection (Supplementary Information 1), we identified a plausible range for β_CC_ = (2.91*10^–5^, 3.05*10^–5^]. This range yielded medians of 24 and 7,753 for infectious birds and living crows at the end of winter, 49 as the median peak daily number of dead birds, and 35% of the 300 simulations met our criteria for realistic outbreaks (Table 2).

**Table 2:** Summary of results for the three outcomes of interest: infectious crows and living crows at the end of winter (day 151), and maximum number of dead birds during the study period, after 300 simulations conducted with parameter values randomly selected from the quartiles for β_CC_ and the ranges for the other 25 parameters **κ**_2–26_.

**β**_**CC**_ **range**	**Outcome of interest**	**Median (Min - max)**	**Proportion of realistic simulations**
[2.48 * 10^−5^_,_ 2.62 * 10^−5^]	Infectious crows last day	2 (0–135)	0.25
	Living crows last day	8,542 (6,105–8,859)	
	Maximum number of dead birds	28 (7–145)	
(2.62 * 10^−5^, 2.77 * 10^−5^]	Infectious crows last day	5 (0–168)	0.31
	Living crows last day	8,406 (5,060–8,849)	
	Maximum number of dead birds	31 (6–264)	
(2.77 * 10^−5^, 2.91 * 10^−5^]	Infectious crows last day	9 (0–180)	0.32
	Living crows last day	8,268 (4,737–8,834)	
	Maximum number of dead birds	37 (9–268)	
(2.91 * 10^−5^, 3.05 * 10^−5^]	Infectious crows last day	24 (0–190)	0.35
	Living crows last day	7,753 (4,444–8,823)	
	Maximum number of dead birds	49 (9–313)	

**Figure 2:**
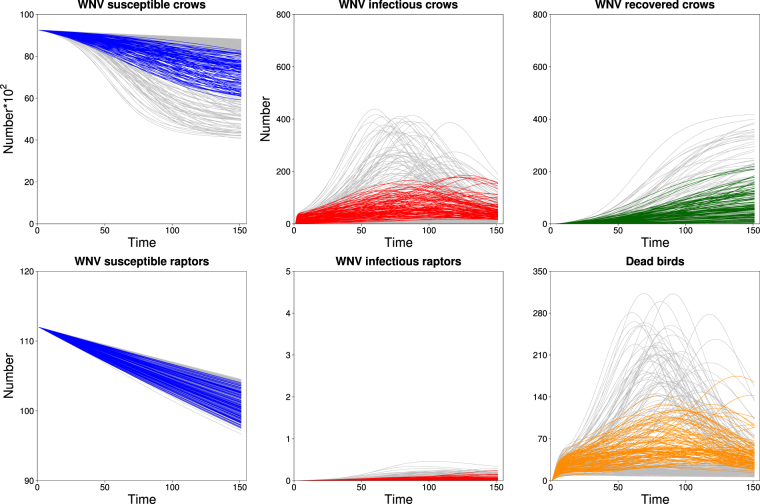
Time series for the number of susceptible, infectious, and recovered crows, susceptible and infectious raptors, and dead birds for each simulation based on random draws from the final selected ranges of β_CC_ and other parameters. Lines represent individual simulations that were either realistic (colored) or unrealistic (gray) based on our defined criteria.

Simulated trajectories over the study period for S_C_ (susceptible crows), the sum of I_C1_, I_C2_ and I_C3_ (infectious crows), R_C_, S_R_, the sum of of I_R1_, I_R2_ and I_R3_ (infectious raptors) and the sum of D_C_, D_R_, D_IR_ and D_O_ (dead birds) are shown in Fig. 2. The realistic simulations, in general, showed slow decline of susceptible crows and a smooth increase in the number of infectious crows over the study period, reaching a median number of 7,238 and 37 of these individuals, respectively, at the end of the winter. The median number of R_C_ (immune crows) by March 31^st^ in successful simulations was 60. The total number of infectious birds consisted primarily of crows. Consequently, the number of dead birds during the study period followed the number of infected crows closely. The median daily number of dead birds for successful simulations was 43. Furthermore, in these realistic simulations the average daily proportion of I_C1_ and I_C2_ (WNV fecal shedders), and I_C3_ (visceral WNV, chronically infected birds) in the roost was 0.005 and 0.0005, respectively. Conversely, simulations that turned out to be unrealistic generally led to early, rapid rises in infected crows and rapid depletion of susceptible crows. The dynamics of raptor infections differed little between realistic and unrealistic scenarios.

### Relevance of transmission among communally roosting crows and alternative WNV transmission pathways between birds such as predation and scavenging

The global sensitivity analysis showed that the three OoI were sensitive to (1) the daily per capita WNV crow-to-crow transmission rate (β_CC_), (2) the daily per capita rate at which I_C1_ clear WNV viremia (γ_C1_), and (3) the probability that I_C1_ die due to WNV infection at the end of the acute viremic period (ρ_C_). Moreover, the initial number of WNV-exposed crows introduced at the start of the winter (E_C_) affected the number of living crows at the end of the study period and the maximum number of dead birds. Finally, the number of infectious birds at the end of the winter was sensitive to the daily per capita rate at which E_C_ become acutely viremic (ε_C_), whilst the number of living crows at the end of the study period was sensitive to the daily per capita mortality rate of crows (μ_C_), and the peak daily number of dead birds was sensitive to the daily per capita rate of D_C_ elimination through decomposition (τ_C_). Therefore, the set of parameters to which our OoI were sensitive were defined as **ϴ = **{β_CC_, E_C_, ε_C_, γ_C1_, ρ_C_, τ_C_, μ_C_}. The corresponding estimates of PRCC and the 95% CI are shown in Table 3.

**Table 3:** Partial rank correlation coefficients estimates (PRCC) and 95% confidence intervals for the parameters to which the three outcomes of interest were significantly sensitive.

Outcome of interest	Sensitive parameter	Estimate	95% C.I.
Infectious birds last day	β_CC_	0.273	0.1–0.430
	ε_C_	−0.202	−0.367–0.025
	γ_C1_	−0.951	−0.965–0.930
	ρ_C_	−0.739	−0.810–0.646
Living crows last day	β_CC_	−0.325	−0.476–0.156
	E_C_	−0.793	−0.851–0.717
	γ_C1_	0.963	0.947–0.974
	ρ_C_	0.617	0.493–0.716
	μ_C_	−0.424	−0.560–0.266
Maximum number of dead birds	β_CC_	0.211	0.035–0.375
	E_C_	0.712	0.611–0.790
	γ_C1_	−0.892	−0.923–0.849
	ρ_C_	−0.357	−0.504–0.192
	τ_C_	−0.370	−0.514–0.206

### Conditions supporting realistic outbreaks and infectious birds at the end of the winter across the parameter space

**Figure 3:**
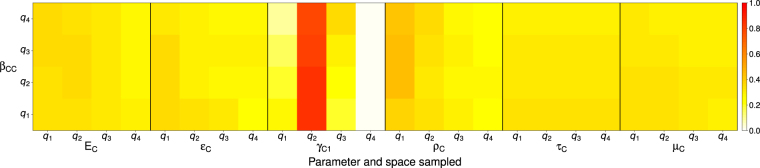
Proportion of simulations that fulfilled our criteria for realistic outbreaks within the joint parameter space defined by quartiles of β_CC_ and each parameter to which our OoI were sensitive.

The proportion of realistic simulations varied little across the ranges of β_CC_ and the ranges of most of the significant parameters, **ϴ** (Fig. 3). The proportion of realistic simulations was maximized at >75% for the middle quartiles of γ_C1_, corresponding to a moderate infectious period in acutely viremic crows that was neither too long nor too short. When the recovery rate of acutely viremic crows was moderately low (*q*_2_), outbreaks most outbreaks were realistic across values of β_CC_, but for high recovery rates (*q*_4_), the opposite was true, yielding no realistic outbreaks (Fig. 3).

## Discussion

We constructed a dynamical model to simulate a bird community consisting of crows, raptors and other birds in order to evaluate whether WNV could persist through the winter under plausible bird-to-bird transmission parameters while causing realistic outbreaks in the absence of mosquito-borne transmission. If WNV is introduced into a completely susceptible bird community at the beginning of winter, our results demonstrate that where they exist, large crow roosts are expected to dominate the dynamics of WNV during the cold season and that plausibly low transmission rates within the roost could enable WNV persistence through the winter. Viremic crows at the end of winter could then initiate horizontal bird-mosquito transmission in the spring when the weather warms and mosquitoes become active.

Our simulations introduce WNV into a completely susceptible communal crow roost at the start of winter. This is a reasonable initial condition because the immune fraction is expected to be low due to the birth pulse of new susceptible crows each spring and death of most infected crows before becoming immune. Because of fecal WNV shedding in highly viremic crows, fecal-oral transmission due to fecal stain and preening behavior is likely the primary crow-to-crow transmission pathway^29,50^, and for this reason we included WNV viremic and non-viremic fecal shedder crows (I_C1_ and I_C2_)_,_

To our knowledge, estimates of bird-to-bird per-capita transmission rate have not been published for WNV in free-ranging birds, but our estimated range for the crow-to-crow daily transmission rate β_CC_ = (2.91*10^–5^, 3.05*10^–5^], which implies 1 WNV transmission event every ~3 days in the initial roost, is within the range of previously published estimates of daily transmission rates for infectious diseases in captive birds and non-roosting wild birds. For example, our range for β_CC_ was lower than the estimated daily per capita transmission rate for WNV in experimental caged crows^79^, for avian influenza in high-density enclosures of poultry^80–82^ and for primarily fecal-oral transmitted bacterial pathogens, such as *Campylobacter C. jejuni*, *C. coli Salmonella enterica* serovar Enteritidis, and *Salmonella* sp. in poultry as well^76,83–86^. In the case of non-communally roosting free-ranging aquatic birds, the estimated daily per capita transmission rate for avian influenza was lower than our selected β_CC_ range^75^ which would be expected.

The number of total infectious birds in the community was heavily driven by crows, and all of the parameters to which our OoI were sensitive to were related to infection dynamics in crows. The duration of fecal shedding following the acute viremia and the duration of the chronic infection had little effect, probably due to the reduced number of crows reaching that stage, as most infected crows die after the acute viremic period^39,40,45–52^. The trajectory of crows in realistic simulations is consistent with results from previous studies. For example, the rarity of seropositive crows is attributable in part to high mortality rates following infection, and one serosurvey did not find seropositive crows during the cold season (February and March 2002) after the initial introduction of WNV in 2001^48^. In our successful simulations the average daily fraction of R_C_ (immune crows) was 0.003. Under this average seroprevalence, sampling zero seropositive crows if collecting blood from 1 to 152 individuals (the last number is the total N reported by the authors of the serosurvey) has a probability between 0.63 to 1. Other published studies have also found low percentages of seropositive crows during the cold season^57,87^.

Moreover, a previous field study of the same roost that we simulated collected 909 WNV-negative fecal samples without finding a WNV-positive sample during the cold season^29^. The authors reported that the probability of such a result, assuming WNV prevalence of 0.023 and independence of observations over time, was <1 * 10^−7^. In our realistic simulations the average daily proportion of crows shedding WNV in feces (I_C1_ and I_C2_) at the roost was 0.005. If we apply this shedding prevalence and assume that: (1) crows defecate once daily at the roost, (2) 88 fecal samples are collected in a single day (the reported median number of samples collected by month during the field study), and (3) samples collected in a single day are independent, the binomial probability of all samples negative is 0.64, which is more consistent with the previous field results^29^. Further, the authors of this same field study also reported 12 WNV positive crow carcasses out of the 32 that were collected under the roost (prevalence of 0.375). In our model, we did not track infectious crow carcasses, however, simulations without any WNV-infected bird introduced caused an average daily number of 7.49 dead crows, while realistic simulations under the selected β_CC_ for WNV transmission yielded 43.03 average daily dead crows. This means that on average ~83% of crow carcasses present in a single day are positive to WNV during winter, which results in a probability >0.999 to find at least one WNV-positive carcass if 10 dead crows are collected in a single day. Moreover, if the 32 carcasses were collected in one day, the probability of finding at least 12 positives is also >0.999. Another study in winter crow roosts was also more successful in finding WNV-infected specimens when testing crow carcasses compared to feces^57^. Finally, because only a small fraction of total dead birds are observed^88^, the average number of dead birds, mainly crows, per day in successful simulations is consistent with observations during WNV outbreaks in initially naive bird communities^89–91^.

Other bird-to-bird transmission pathways, including scavenging of crows upon other birds and predation by raptors, were not relevant for WNV dynamics or the persistence of the virus in the bird community during the winter. These phenomena may be due to the relatively small number of raptors within the avian community studied, which limited the overall rate of contact between raptors and crows. These results are consistent with previous model-based findings that transmission between crows via close contact could have a considerable impact on WNV establishment when the density of ornithophilic mosquitoes is low and that during such periods, scavenging and the effects of other birds in the community are not relevant for determining the WNV basic reproductive number (R_0_)^79^. Furthermore, current data show that many infected raptors do not develop WNV infection in the organs or shed the virus through their feces^39,59^. This would further diminish the importance of this group of birds for WNV dynamics.

The realism of outbreaks was remarkably dependent on the value of the infectious period of acutely viremic crows. Infectious periods that were too short (3.0–3.3 days) or too long (4.3–5.0 days) resulted in very few or not realistic outbreak trajectories over the winter, whereas moderate acute infectious periods of 3.3–4.3 days were much more realistic, specially in the range 3.7–4.3 days.

Future modelling work should consider the effects of stochasticity and different population sizes of the crows, raptors, and other birds within the community. Our model assumes that crow density declines in proportion to the total number of crows in the roost, but future studies should consider whether crow mortality also results in changes to aggregation and roost structure that may affect density. Models should also account for the fact that crows have variable home range sizes^92^ and that infected crows may roost with susceptible crows less frequently during illness associated with acute viremia^93^. Potential effects of temperature on crow behavior and resulting differences in contact rates should also be considered. Evaluating these additional aspects will determine the generalizability of our results to other epidemiological settings and also to study the role of crows in WNV spread and maintenance across space and time.

## Conclusions

Our results strongly support plausible scenarios by which birds can sustain WNV through the winter via bird-to-bird transmission, specifically due to a limited but persistent number of new WNV crow-to-crow transmission events. In nature, these transmission events would be driven mainly by WNV fecal shedding by infectious crows, fecal staining of susceptible individuals, and posterior preening. The values for the daily per capita WNV crow-to-crow transmission rate are sufficiently small to be plausible when density-dependent transmission is assumed. Moreover, these values are between estimates for pathogen transmission parameter in poultry and in non-roosting wild birds. The realistic simulations generate consistently low numbers of infectious and recovered crows over the study period and a higher number of dead crows in the system compared to periods when WNV is absent. These characteristics are consistent with a high WNV detection probability in crow carcasses found under roosts during winter, a low probability of WNV detection in feces during the same period, and low seroprevalence in the population. Our findings add to previous research on the importance of crow roosts for WNV overwintering and WNV dynamics in crows and improve our understanding on how WNV persists in temperate climates when cold temperatures preclude viral replication and diminish vector blood-feeding activity.

## Electronic supplementary material

Supplementary Table
