# Supplementary material for: Overwintering of West Nile virus in a bird community with a communal crow roost
Source: Sci Rep. 2018 Apr 17;8:6088. doi: 10.1038/s41598-018-24133-4 (PMC5904116; doi:10.1038/s41598-018-24133-4)
Supplement: Supplementary file 1 — Supplementary Table [file 41598_2018_24133_MOESM1_ESM.pdf]

Title: Overwintering of West Nile virus in a bird community with a communal crow roost

Authors: Diego Montecino-Latorre[1,*]; Christopher M. Barker[2]

[1]One Health Institute, School of Veterinary Medicine, University of California Davis, One Shields Ave., Davis, California 95616, USA.
[2]Department of Pathology, Microbiology, and Immunology, School of Veterinary Medicine, University of California, Davis, CA 95616, USA.

* Corresponding author: dmontecino@ucdavis.edu

Supplementary table. Summary of results for the three outcomes of interest: infectious birds at day 151, alive crows at day 151 and maximum number of dead birds during the study period, after 300 simulations conducted with parameter values stochastically selected with LHS from ranges of values for κ_2,...κ_{26} and β_{cc}.

β_{CC} range	Outcome of interest	Median (Min- max)	Proportion of successful simulations
$2.14*10^{-9}$ – 2	Infectious birds last day	13 (0 - 54)	0
	Alive crows last day	439 (0 - 876)	
	Maximum number of dead birds	2,921 (1,970 – 3,985)	
$2.14*10^{-9}$ - 0.5	Infectious birds last day	13 (0 - 54)	0
	Alive crows last day	437 (3 - 888)	
	Maximum number of dead birds	2,374 (1,505 – 3,406)	
$2.14*10^{-9}$ - 0.125	Infectious birds last day	13 (0 - 51)	0
	Alive crows last day	436 (1 - 880)	
	Maximum number of dead birds	2,365 (1,433 – 3,409)	
$2.14*10^{-9}$ - 0.0315	Infectious birds last day	13 (0 - 51)	0
	Alive crows last day	441 (1 – 1,100)	
	Maximum number of dead birds	2,360 (1,095 – 3,510)	
$2.14*10^{-9}$ - $8*10^{-3}$	Infectious birds last day	14 (0 - 56)	0
	Alive crows last day	446 (3 – 8,417)	
	Maximum number of dead birds	2,334 (18 – 3,341)	
$2.14*10^{-9}$ - $2*10^{-3}$	Infectious birds last day	164 (0 - 74)	0.003
	Alive crows last day	461 (1 -8,861)	
	Maximum number of dead birds	2,244 (7 – 3,182)	

$2.14*10^{-9}$ - $5*10^{-4}$	Infectious birds last day	12 (0 - 71)	0.0067
	Alive crows last day	562 (3 – 8,840)	
	Maximum number of dead birds	1,936 (8 – 3,215)	
$2.14*10^{-9}$ - $1.22*10^{-4}$	Infectious birds last day	13 (0 - 115)	0.0467
	Alive crows last day	1571 (134 – 8,880)	
	Maximum number of dead birds	764 (5 – 2,395)	
$2.14*10^{-9}$ – $3.05*10^{-5}$	Infectious birds last day	0 (0 - 249)	0.0767
	Alive crows last day	8,708 (4,599 – 8,876)	
	Maximum number of dead birds	21 (5 -196)	
$2.14*10^{-9}$ – $7.63*10^{-6}$	Infectious birds last day	0 (0 - 0)	0
	Alive crows last day	8,756 (8,627 – 8,878)	
	Maximum number of dead birds	14 (4 - 28)	
$3.05*10^{-5}$ - $1.22*10^{-4}$	Infectious birds last day	18 (0 – 110)	0.0233
	Alive crows last day	1,072 (115 – 8,163)	
	Maximum number of dead birds	1,053 (18 – 2,304)	
$7.63*10^{-6}$ - $3.05*10^{-5}$	Infectious birds last day	16 (0 – 134)	0.08
	Alive crows last day	8,689 (4,616 – 8,871)	
	Maximum number of dead birds	16 (5 – 169)	
$7.63*10^{-6}$ - $1.335*10^{-5}$	Infectious birds last day	0 (0 - 47)	0
	Alive crows last day	8,742 (8,598 – 8,874)	
	Maximum number of dead birds	14 (4 - 34)	

$1.335*10^{-5}$ - $1.907*10^{-5}$	Infectious birds last day	0 (0 – 4)	0
	Alive crows last day	8,721 (8,454 – 8,871)	
	Maximum number of dead birds	15 (5 – 32)	
$1.907*10^{-5}$ – $2.48*10^{-5}$	Infectious birds last day	0 (0 – 77)	0.07
	Alive crows last day	8,648 (7,392 – 8,866)	
	Maximum number of dead birds	17 (5 – 54)	
$2.48*10^{-5}$ - $3.05*10^{-5}$	Infectious birds last day	7 (0 - 176)	0.3
	Alive crows last day	8,340 (4,290 – 8,844)	
	Maximum number of dead birds	24 (4 - 275)	
$3.05*10^{-5}$ – $5.3*10^{-5}$	Infectious birds last day	23 (1 - 107)	0.12
	Alive crows last day	3,739 (1,345 – 8,729)	
	Maximum number of dead birds	290 (11- 1,037)	
